# Physical attractiveness, same-sex stimuli, and male venture capitalists’ financial risk-taking

**DOI:** 10.3389/fpsyg.2023.1259143

**Published:** 2024-01-12

**Authors:** Marc D. Bahlmann

**Affiliations:** VU Amsterdam, Amsterdam, Netherlands

**Keywords:** venture capital, financial risk-taking, same-sex stimuli, physical attractiveness, bias

## Abstract

Financial risk-taking is central to venture capital decision-making, which is increasingly approached from a heuristics and biases perspective. While previous research has identified entrepreneurs’ physical attractiveness as an important heuristic cue in VCs’ investment decisions, this study addresses the role of VCs’ own physical attractiveness in relation to the financial risks they take. Using a dataset for a representative sample of 341 male entrepreneur and male VC dyads in the context of stage financing, this study finds that VCs of below-average attractiveness are more sensitive to the physical attractiveness of the entrepreneur when compared to VCs of average attractiveness. Also, the nature of this effect changes from the first to the second investment round for VCs of below-average attractiveness. Combined, these findings imply that VCs’ funding decisions may be subject to mechanisms that stem from their own physical attractiveness. Theoretical implications for VC decision-making and same-sex stimuli are discussed.

## 1 Introduction

Opposite-sex stimuli are a known and well-documented factor in shaping people’s judgments and behaviors, among which the likelihood of accepting unfair offers, being more tolerant of ethically ambiguous behaviors, the preference for riskier strategies in chess, the likelihood of making charitable donations, and taking financial risks (Wilson and Daly, 1985; Ariely and Loewenstein, 2006; Landry et al., 2006; Baker and Maner, 2008; Bertrand et al., 2010; Dreber et al., 2013). Recent research has started to consider the potential effects of *same-sex* stimuli on judgments and behaviors as well. Nonetheless, there still is a need for research on whether and how same-sex stimuli also impact people’s judgments (Chan, 2015). To address this gap, Chan (2015) showed across four experiments how physically attractive males impact men’s financial risk-taking, and offered a rationale for this effect by building on social-comparison and fluid-compensation theory. In short, as men have faced greater intrasexual competition in attracting women as a mating partner throughout evolutionary history, the average heterosexual man who perceives a male counterpart to be more physically attractive than he is (i.e., the social comparison-part), should be motivated to increase his desirability in other ways (i.e., the fluid compensation-part). An effective way to increase one’s desirability is by accruing more financial assets, so as to compensate for a perceived lack in physical attractiveness. Taking more financial risks has been proposed as an effective strategy to achieve this.

To date, same-sex effects on male financial risk-taking have only been demonstrated in an experiment-based setting and, thus, have not been shown to account for variation in judgments and behaviors in naturalistic settings. The current study explores and extends this line of research in the context of venture capital funding decisions. Venture capitalists (VCs) can be defined as professional investors who fund portfolios of ventures with high-growth potential (Drover et al., 2017), and in so doing incur substantial financial risks. While earlier research predominantly assumed VCs to act rationally in their screening, selection, and funding of new, high-technology ventures, this assumption has increasingly been met with skepticism (e.g., Franke et al., 2006; Matusik et al., 2008; Murnieks et al., 2011). VC investor decisions have particularly been found to be sensitive to the physical attractiveness of ventures’ lead entrepreneurs, both in the context of venture screening (Baron et al., 2006; Brooks et al., 2014) and venture funding (Bahlmann, 2023). These studies suggested that VCs are susceptible to the attractiveness halo when assessing the riskiness and potential of a new venture. The attractiveness halo is a type of cognitive bias that captures the human tendency to assume that physically attractive individuals possess positive qualities beyond their physical appearance (Langlois et al., 2000). In the context of VC selection and funding, physically attractive entrepreneurs seem to be attributed desirable personality traits by VCs – such as intelligence, trustworthiness, and leadership qualities – that influence their risk assessments of associated ventures (Baron et al., 2006; Brooks et al., 2014). As both the high-technology venture setting and the venture capital industry are male dominated (Atomico, 2020), the high probability of male same-sex encounters may introduce comparison-based and compensatory mechanisms in addition to the halo effect. Indeed, the attractiveness of the VC himself in relation to his risk estimations and risk-taking has yet to be considered. As such, the role that physical attractiveness plays in the VC context has been approached from one perspective only, and hence may not be fully understood at present.

This study not only intends to validate the generalizability of earlier, experiment-based research in a naturalistic setting, but also seeks to extend this line of research by considering the effect of same-sex stimuli on financial risk-taking beyond the initial agent-target encounter. To date, studies on same-sex stimuli and financial risk-taking have considered one-time agent-target encounters, where the agent and target had no actual relationship with one another. Whether same-sex stimuli effects linger on beyond initial encounters is unclear yet important to understand, for financial risk-taking decisions tend to materialize over time and can be of a recurring nature (Sahlman, 1990; Gompers, 1995).

This study therefore aims to determine how the physical attractiveness of male entrepreneurs impact male VC financial risk-taking as a function of their own physical attractiveness, and does so by utilizing VC funding data of 341 European IT ventures. Particularly, this study explores the role of VC attractiveness in the context of VC stage financing, which involves the gradual funding of ventures and adjusting or aborting potential subsequent investments as more insight into a venture develops.

## 2 Theoretical background

### 2.1 VC risk assessment and funding behavior from an agency perspective

VCs raise funds to build portfolios of high-growth, high-risk ventures and provide mentorship and monitoring to support their success (Sahlman, 1990; Hellmann and Puri, 2002). The ultimate goal of VCs is to exit these ventures and generate favorable returns. This pursuit is not without challenges, as VCs encounter substantial information asymmetry and uncertainty (Sahlman, 1990; Gompers, 1995; Gompers and Lerner, 2001). Early-stage ventures typically lack performance history, operate in highly unpredictable and competitive environments, and often experience many years of negative earnings (Wang and Zhou, 2004; Li, 2008). Consequently, most VCs opt for stage financing (also known as staging) as the preferred funding approach for the ventures they selected. This funding approach is recognized as a key mechanism to control the venture (Sahlman, 1990). VCs prefer stage financing because they are confronted with a separation of ownership and control that generates so-called agency costs for the VC (Tian, 2011). These costs call for mechanisms that enable the VC (i.e., the principal) to regulate harmful expenses caused by the entrepreneur (i.e., the agent). For example, entrepreneurs may be inclined to overspend on research and development (R&D) or hastily introduce a product to the market (Gompers and Lerner, 2001; Tian, 2011). VCs strive to minimize any information asymmetries and agency costs to ensure that the venture receives the appropriate funding based on its actual potential. Staging is viewed as an effective and efficient method to reduce or prevent anticipated agency costs by keeping entrepreneurs under strict supervision (Gompers and Lerner, 2001).

Despite its advantages, staging has several drawbacks as well in the form of monitoring costs. Staging requires significant time and effort from VCs, who need to attend board meetings, visit venture sites, and participate in day-to-day operations to successfully monitor and enhance venture performance (Sahlman, 1990; Kaplan and Strömberg, 2003; Bottazzi et al., 2008). Additionally, each staging round necessitates additional negotiation and contracting efforts, as well as resources and time to evaluate a venture’s progress and prospects (Kaplan and Strömberg, 2003). Furthermore, staging can incentivize entrepreneurs to depict their ventures’ progress too optimistically or focus on short-term gains at the expense of long-term value creation (Tian, 2011). Lastly, staging can lead to delays in venture development by underinvesting in the initial development phase (Wang and Zhou, 2004). Therefore, VCs are expected to consider the costs of staging in relation to expected agency risks. Accordingly, VCs seek the most effective staging strategy to align their interests with those of the entrepreneur, and in view of perceived risks and opportunities. Prior research found that ventures that are viewed as more risky by the VC, on average receive smaller amounts of funding per staging round to discipline the entrepreneur and increase VC control (Gompers, 1995; Li, 2008; Tian, 2011). In these instances, VCs are willing to accept more monitoring costs in view of anticipated agency issues. When ventures are perceived as less risky, funding amounts tend to be higher per staging round on average, implying less stringent control by the VC and more room to maneuver for the entrepreneur. From a VC perspective, the benefit of offering higher amounts per staging round and associated maneuverability lies in preventing the entrepreneur from creating a deceptively favorable impression of the venture (also referred to as window-dressing) while enabling the venture to develop economies of scale (Tian, 2011). As such, VCs’ preferred staging approaches are assumed to reflect underlying risk estimations that are based on rational and efficiency-driven considerations (Gompers, 1995).

While agency theory helps to interpret VCs’ preferred staging approaches in terms of risk judgment and perception, it does not suffice to capture the underlying intuitive nature of how VCs arrive at their risk assessments (Bahlmann, 2023). By approaching VC staging as rational decision-making contexts, emphasis has been put on System 2 thinking, which is a mode of thought that is slow, more deliberative, and more logical. This for instance would be the case when an investor tries to calculate the potential gains of an investment. This may have led to a neglect for the potential presence of System 1 thinking, which is a more intuitive and automatic mode of thought, characterized by more emotional and instinctive decision-making (Kahneman, 2011). System 1 thinking involves the strong reliance on heuristics, which for instance occurs when inferring one’s trustworthiness from his/her physical appearance (Langlois et al., 2000).

### 2.2 VC risk assessment and staging behavior from an evolutionary perspective

From an evolutionary perspective, individuals’ behaviors have evolved in response to adaptive challenges throughout evolutionary history, whereby the human brain is specifically adapted to the ancestral environment in which the human species evolved (Kanazawa and Kovar, 2004). An important adaptive challenge that both men and women face is to achieve reproductive success through mating. Sexual selection leads to adaptations resulting from successful mating (Darwin, 1871), and involves two distinct processes: intersexual selection, which involves mate choice, and intrasexual selection, which involves competition between members of the same sex to gain access to members of the opposite sex (Bateman, 1948; Trivers, 1972; Brown et al., 2009). Men can succeed in intrasexual competition by displaying their underlying mate qualities through easily observable yet difficult to pretend signals (Zahavi, 1975). Examples of such signals are facial hair (Neave and Shields, 2008; Dixson and Brooks, 2013), voice pitch (Puts et al., 2007), and body shape (Coy et al., 2014). Additionally, certain male behaviors, such as intrasexual aggression (Daly and Wilson, 2001; Archer, 2009) and conspicuous consumption practices to display wealth (Saad, 2007) have also been suggested to be signals shaped by sexual selection.

The signals that heterosexual men display, are directed at heterosexual women. When looking for potential partners to mate with, women first of all consider a male’s physical attractiveness as a highly desirable feature (Landolt et al., 1995). Physical attractiveness is signaled through both bodily and facial features, such as muscular strength, body height and facial attractiveness. These features may signal underlying qualities such as masculinity, dominance, and health, and assist women in assessing general mating quality (Penton-Voak and Perrett, 2000; Frederick and Haselton, 2007). It is important to note, however, that male attractiveness influences females’ self-perceived physical attractiveness and their preferences for masculinity and dominance (Little and Mannion, 2006). Besides using physical attractiveness as a heuristic cue for underlying mating qualities, women also look for signs of relational commitment, intellectual capability, and other skills, so as to deal with their adaptive problem of nurturing their offspring (Townsend and Levy, 1990; Buss and Schmitt, 1993). A male’s economic status functions as such an adaptive cue. Previous studies have, for example, demonstrated that men’s reproductive success is a function of their economic position (Hill and Hurtado, 1996; Hopcroft, 2006). Also, women have been found sensitive to a male’s monetary income when browsing through personal ads (Campos et al., 2002; Pawlowski and Koziel, 2002). The two adaptive cues that women look for in a potential mating partner enable men to compensate for sub-optimal performance in either the attractiveness- or economic status-domain. Following compensatory theories in psychology, a perceived lack of attractiveness should motivate men to compensate for this deficiency by increasing their desirability in another way (Salthouse, 1995). In particular, men will be motivated to increase their desirability, such that they achieve the same higher-level goal (that is, appearing to be a desirable mating partner) (Tesser, 2000; Chan, 2015). In the absence of physical attractiveness, increasing one’s financial position by pursuing more risky yet potentially highly prosperous opportunities is a likely alternative strategy.

Prior research suggested that the two heuristic cues that women use to select a potential mate affect males’ inclinations to take financial risks in a context of same-sex stimuli as well. Chan’s, 2015 study was among the first to specifically look into the role of attractiveness in heterosexual male-to-male encounters. Across four experiments, Chan concluded that “*men who see attractive males take greater financial risks than those who do not (…) when (1) they perceive their physical attractiveness to be lacking (…), (2) they have a lower income than the average American man (…), and (3) they have a mating motive that heightens their instinct to increase their desirability as a mating partner to women*” (Chan, 2015: 412). As Chan (2015) asserts, an average heterosexual man who sees an attractive male is likely to perceive himself as less physically attractive and desirable as a mating partner to women (Kenrick and Guttierres, 1980; Thornton and Moore, 1993). Seeing an attractive male may incite a man’s motivation to increase his desirability, prompting him to accrue more financial resources by taking more financial risks. Chan’s rationale is echoed in studies of male conspicuous consumption patterns and how these males are viewed by other men in terms of mate value characteristics such as attractiveness, status, and ambition (Hennighausen et al., 2016). From this perspective, the signaling of economic status by the conspicuous consumption of luxurious goods or expansive brands is considered a difficult to pretend signal of underlying desirable traits (Miller, 2009; Nelissen and Meijers, 2011).

For this study, the effect of VC physical attractiveness on VC risk-taking is anticipated to manifest in two ways. With regard to the first way, it is expected that VCs of above-average attractiveness are more tolerant of the risks associated with investing in new ventures when compared to VCs of below-average attractiveness. Earlier research has demonstrated attractive people to have more positive risk attitudes (Refaie and Mishra, 2020), and has also showed that people who consider themselves physically attractive have higher self-esteem (Thornton and Ryckman, 1991; Bale and Archer, 2013). Self-esteem has subsequently been positively associated with trust in others (Smith et al., 2009) as well as financial risk tolerance (Grable and Joo, 2004) and various types of financial behavior (even in the presence of objective financial knowledge (Tang and Baker, 2016)). People who score high on self-esteem have also been found to have a higher propensity to invest and take investment risks (Sekścińska et al., 2021). At the same time, people with relatively lower levels of self-esteem have been found to respond negatively to ambiguous information, which often is the case in venture capital decision-making (Stanovich and West, 2000; McElroy et al., 2007). Following this, VCs of above-average attractiveness are generally expected to take more risks compared to VCs of below-average attractiveness, as manifested by higher average amounts of funding in the first rounds of venture investment.

With regard to the second way, it is expected that VCs’ responses to entrepreneurs’ attractiveness will differ between VCs of above-average and below-average attractiveness, such that VCs of below-average attractiveness are more sensitive to the attractiveness halo effect. First, following the argument developed by Chan that builds on social comparison and fluid compensation theory, VCs of below-average attractiveness are more likely to perceive themselves as less physically attractive, and therefore are more likely to show compensation-behavior by taking financial risks. Second, VCs of below-average attractiveness are more likely to experience a threat to self-esteem. Whereas low self-esteem people have been found to respond more negatively to same-sex attractive individuals (Agthe et al., 2010), previous research also indicated that people experiencing a threat to self-esteem become more reliant on stereotyping, which in this case could make VCs more susceptible to the attractiveness halo (Westfall et al., 2020). To conclude, physically attractive VCs are anticipated to take more risks in general in the early stages of the funding process, but at the same time are less susceptible to the physical attractiveness of the venture’s lead entrepreneur compared to VCs of below-average attractiveness.

## 3 Methods

### 3.1 Research setting and sampling

The objectives of this research were pursued in the European IT industry, which represents a male-dominated environment with many high-tech IT ventures characterized by asset-intangibility and high market-to-book ratios (i.e., high risk) on the one hand, and a well-developed venture capital sector on the other (Atomico, 2020). Data collection took place in the first half of 2017. As a first step, a sample of IT ventures was compiled by consulting two online platforms that keep track of venture funding processes and associated entrepreneurs and investors, namely AngelList and Crunchbase. To increase data quality and reliability, ventures were sampled only if their online profiles contained information on entrepreneurial team composition, funding process, and associated investors on both platforms. Moreover, additional LinkedIn profiles for associated ventures, entrepreneurs, and investors had to be present to facilitate the collection of venture-level and individual-level data. Overall, 609 IT ventures met these sampling criteria. The representativeness of the sample was checked as follows. First, the geographic distribution of the sampled ventures across European cities was compared to the distribution of funded ventures for the entire European population (based on Atomico, 2018). Second, the distribution of the capital disbursement amounts for the sampled ventures were compared to that of the European population of ventures (Atomico, 2018). The sample appeared to match the qualities of the general population based on face validity.

The sample of 609 IT ventures was brought back to 341 during the analysis process for two reasons. First, as the study focuses on same-sex stimuli, only male-based venture-investor dyads were included. Whenever an entrepreneurial or investor-team contained one or more females, the venture was excluded from analysis so as to prevent any opposite-sex stimuli from affecting VC judgement. Consistent with the general underrepresentation of women in the IT- and VC industries, only nineteen ventures were excluded. Second, as the study focused on both VC physical attractiveness and entrepreneurial attractiveness, LinkedIn profile photo’s for both the lead-entrepreneur and investor had to be present for each venture that would allow valid attractiveness ratings (see data collection procedure below). It turned out that for 341 ventures, profile photo’s for both the lead investor and lead entrepreneur were available. These 341 ventures were subsequently included in the analysis. T-tests revealed that the included ventures did not systematically differ from the 268 that were excluded in terms of firm age (*p* = 0.673), team size (*p* = 0.628), educational diversity (level and type) (*p* = 0.230 and.294 respectively), and geographic distance (*p* = 0.960).

### 3.2 Data collection procedures

The data for this study were independently hand-collected by two researchers because the research questions cannot be addressed with existing commercial databases (Hellmann and Puri, 2002; Bottazzi et al., 2008). All data-entries were cross-verified. The first step involved collecting venture-specific data from AngelList, Crunchbase, LinkedIn, VC websites, and corporate websites. These data sources were used to gain insight into venture funding amounts, the names of the entrepreneurs and investors involved, number of employees per venture, and office locations of both the venture and VC firm.

The second step involved collection data about the 1,486 entrepreneurs and 2,578 investors associated with the 609 ventures. Demographic information in terms of educational background, gender, professional experience was collected through LinkedIn. This step also served to identify the lead entrepreneur and lead investor for each venture. In case more than one entrepreneur was associated with a venture, he who would identify himself as CEO and founder on LinkedIn would be considered the lead entrepreneur. The lead investor was identified by selecting the most experienced investor who had been with the venture from the very start of the funding process (Bahlmann, 2023).

The third step of data collection involved estimating entrepreneurs’ and investors’ physical attractiveness. To this end, their LinkedIn profile photos were rated by human raters (Anderson et al., 2001; Little et al., 2011), which is detailed below.

### 3.3 Measures

#### 3.3.1 Dependent and independent variables

To capture VCs’ financial risk-taking, this study relied on two specific metrics to consider whether and how VCs’ physical attractiveness mattered in response to entrepreneurs’ attractiveness. First, the study utilized the amount of funding during the first round of investment to capture *first-round risk-taking*. Previous research has indicated that funding amounts are good indicators of VC risk perception (Tian, 2011). Second, the second round of investment was used to calculate the relative increase or decrease in investment after the first staging round. This was done by dividing the second-round investment by the first-round investment, thereby providing an indication for *second-round risk-taking*. First-round investments are available for all 341 included ventures, of which 172 ventures were able to secure a second-round investment.

To determine entrepreneurs’ and VC investors’ physical attractiveness, student raters were employed to rate all available profile photos in response to the questions ‘How attractive is the entrepreneur?’ or ‘How attractive is the investor?’ respectively. The accompanying Likert scale ranged from 1 (very unattractive) to 7 (very attractive). The inter-rater reliability of physical attractiveness was 0.92 (*p* < 0.001). Ten graduate student raters [five females and five males with different ethnic backgrounds and an average age of 22.8 (SD.74)] were employed to rate the physical attractiveness of each entrepreneur and VC investor in the dataset. Each rater rated the full list of individuals in randomized order to prevent systematic sequential bias (Kondo et al., 2012), and were instructed to work for no more than one hour a day on the task to ensure optimal concentration.

#### 3.3.2 Controls

This study employs several control variables to limit omitted variable bias. To control for venture-specific characteristics, this study controls for *venture age* at the first round of investment, because longer venture track records can impact VCs’ risk assessments. *Employee growth* was incorporated to capture differences in success and, potentially, venture quality and scalability. To this end, the number of employees at the time of data collection was divided by the number of years a firm had been in existence. The study also controls for startups having a business-to-business (B2B) or business-to-consumer (B2C) orientation (1 for B2B), as VCs’ agency considerations are likely affected by such orientations (Bahlmann, 2023).

To control for differences in venture team-based and entrepreneurial qualities, the study first of all controls for *venture team size* by including the number of entrepreneurs associated with the venture at its start. Team size may affect both team functioning and VC valuation (Murnieks et al., 2011; Mao et al., 2016). To control for additional differences in venture team-characteristics, *venture team educational diversity* (in terms of level and type)^1^ were controlled for, as educational diversity has been found to impact external capital providers’ tendency to provide capital (Vogel et al., 2014). Also, the number of days of *entrepreneurial experience* of a venture’s entrepreneurial team at the time of the first investment round was included in the analysis, for experience is an important risk indicator from a VC perspective (Hsu, 2007). Given this study’s focus on the lead entrepreneurs’ physical attractiveness, a *venture team physical attractiveness* control was incorporated for the physical attractiveness of the other team members as well.

Given this study’s focus on the VC investor, a third group of controls was included to capture additional VC investor differences that could be relevant to their risk-taking. First, *VC investor team size* was included to control for syndication as a way risk mitigation (Sahlman, 1990). Additionally, *VC investor team educational diversity* (both level and type, see Footnote 1) was included as diversity of perspectives and backgrounds may affect both risk-taking propensities and quality of discussions (Hambrick and Mason, 1984). *Investor VC experience* was included by calculating the average number of years of VC experience of all investors associated with a given venture at the time of first investment. Controlling for this is of relevance, for experience has been associated with risk-taking propensity (Hsu, 2007). Finally, the *number of offices* a VC’s portfolio company had at the time of investment was included to capture potential differences in portfolio size, resources, capabilities, and associated support structures.

A fourth group of controls was included to capture dyadic qualities of the VC – entrepreneur relationships that form the unit of analysis in this study. *Educational similarity* and *co-ethnicity* were included to control for potential similarity effects (Franke et al., 2006). For educational similarity, a dyad would receive a 1 if the educational backgrounds of both the lead VC and lead entrepreneur fell within the same category (e.g., Business & Economics), and a 0 if otherwise. In line with several other studies (i.e., Webber, 2007; Kerr, 2008; Hegde and Tumlinson, 2014; Bengtsson and Hsu, 2015), co-ethnicity was established by means of assigning entrepreneurs and investors to ten pre-specified ethnic categories based on their surnames. Several robustness checks were performed to ensure sound specification of one’s ethnic background. First, entrepreneurs’ and investors’ front names were used to limit flaws resulting from some surnames being common in more than one ethnic group. For instance, the surname ‘Lee’ occurs among Anglo-Saxons as well as Asians. But an individual called ‘Andrew Lee’ is more likely of Anglo-Saxon origin, while ‘Hua Lee’ is more is more likely of Asian descent. Second, 150 individuals from the sample population were contacted by telephone to verify their ethnic origin. 73 individuals participated in a brief interview, of whom 72 verified the ethnic category assigned to them. One individual identified with a different ethnic category, and two respondents identified with more than one ethnic category. A dyad was assigned a 1 in case both the lead entrepreneur and VC investor fell within the same ethnic category, and 0 if otherwise. *Geographic distance* (log) was included to capture the actual kilometric distance between the lead VC investor and the venture firm based on country of origin, because previous research found geographic distance to generate agency costs (Tian, 2011; Bahlmann, 2016).

Because this study relied on unstandardized, impromptu photographic portraits to estimate entrepreneurs’ and investors’ physical attractiveness, a control was included for differences in photo quality as this may affect attractiveness assessments. The earlier mentioned group of ten raters were asked to assess the extent to which they felt that the LinkedIn profile picture enabled a good judgment of attractiveness on a 5-point Likert scale. This led to the inclusion of photo quality controls for the lead entrepreneur, the lead VC investor, and the other venture team members (*photo quality venture team*).

Finally, several controls were included to cover contextual differences. Venture’s location differences were controlled for by incorporating a GDP dummy for relatively small (e.g., Vienna), and relatively large (e.g., Berlin) European cities. As ventures started and received their first funding round in different years, *year dummies* were included in the analyses. Also, *sub-industry dummies* were incorporated to capture different venture market orientations. While the analyses involve IT-ventures only, there’s turned out to be quite some variety in terms of the markets they addressed. To capture differences in market orientation, ventures’ LinkedIn profile descriptions were used to categorize ventures across the following NACE categories: advertising; education; entertainment; financial activities; fitness and health; ICT; manufacturing; real estate activities; retail; professional services; transport; travel.

## 4 Results

### 4.1 Correlations and regression results

Table 1 reports descriptive statistics and correlations of the variables included in the study. The average physical attractiveness of the VC investors was 3.644, with a standard deviation of 0.960. The average lead entrepreneur’s attractiveness was 3.811 (SD = 0.991). The average logged first-round risk-taking (*n* = 341) was 13.917 (SD = 1.264), while the average second-round risk-taking (*n* = 172) was 1.043 (SD = 0.100). Variance inflation factors (VIF) indicated low levels of multicollinearity.

**Table 1:** Means, standard deviations, and correlations.

Variables	Means	SD	1	2	3	4	5	6	7	8	9	10	11	12	13	14	15	16	17	18	19	20	21	22	23
1. First-round risk-taking	13.917	1.264	-																						
2. Second-round risk-taking	1.043	0.100	−0.139	-																					
3. Lead entrepreneur Physical attractiveness	3.811	0.991	0.153^**^	−0.057	-																				
4. Lead investor physical attractiveness	3.644	0.960	0.122^*^	0.091	0.107^*^	-																			
5. Venture age	6.200	1.086	0.360^**^	−0.034	−0.038	−0.006	-																		
6. Employee growth	11.144	23.731	0.300^**^	0.145	0.004	0.152^**^	0.054	-																	
7. B2B-dummy	0.64	0.480	−0.054	−0.114	−0.008	−0.090	−0.015	−0.102^*^	-																
8. Venture team size	2.48	1.153	0.137^**^	0.095	0.056	−0.021	−0.028	0.054	−0.061	-															
9. Venture team educational diversity (level)	0.22	0.379	0.014	−0.032	−0.041	−0.061	0.044	−0.024	−0.070	0.256^**^	-														
10. Venture team educational diversity (type)	0.38	0.434	−0.053	0.009	−0.115^*^	−0.105^*^	−0.002	−0.008	−0.065	0.352^**^	0.267^**^	-													
11. Entrepreneurial experience (days)	3099.481	1471.136	0.127^*^	−0.020	−0.031	−0.007	0.057	0.090	0.031	0.033	−0.032	0.012	-												
12. Venture team ph. Attractiveness	3.498	2.770	0.100^*^	0.097	0.077	−0.010	−0.057	0.032	−0.077	0.951^**^	0.234^**^	0.313^**^	0.006	-											
13. VC Investor team size	5.250	4.564	0.126^*^	0.168^*^	−0.002	0.025	0.071	0.163^**^	−0.050	0.029	−0.051	0.135^**^	−0.097	0.010	-										
14. VC Investor team educational diversity (level)	0.33	0.357	0.091	0.136	0.086	−0.060	0.054	0.094	−0.068	0.102^*^	0.024	0.041	−0.026	0.086	0.288^**^	-									
15. VC Investor team educ.div. (type)	0.37	0.363	0.072	0.085	0.128^*^	−0.011	0.013	−0.011	0.017	0.045	0.060	0.033	−0.134^**^	0.057	0.322^**^	0.427^**^	-								
16. Investor VC experience (years)	4.119	4.814	0.035	−0.212^**^	−0.001	−0.029	0.023	0.057	−0.044	−0.016	−0.032	0.002	0.265^**^	−0.013	−0.111^*^	−0.028	−0.089	-							
17. Number of offices	1.38	0.916	0.177^**^	0.135	−0.104^*^	−0.017	0.095	0.263^**^	0.043	0.108^*^	−0.026	0.003	0.097	0.055	0.077	0.114^*^	0.000	0.007	-						
18. Educational similarity	0.70	0.458	0.039	0.102	0.340^**^	0.054	−0.021	0.066	−0.033	0.055	−0.127^*^	−0.034	−0.048	0.079	0.120^*^	0.017	0.061	−0.007	0.078	-					
19. Co-ethnicity	0.62	0.487	−0.046	−0.049	0.053	0.139^**^	−0.023	−0.004	−0.112^*^	0.009	−0.034	0.052	−0.074	0.024	−0.138^**^	−0.063	−0.052	−0.037	−0.135^**^	0.050	-				
20. Geographic distance (log)	5.984	3.368	0.151^**^	0.104	−0.106^*^	−0.045	0.075	0.133^**^	0.018	0.040	−0.006	0.011	0.065	0.018	0.318^**^	0.207^**^	0.225^**^	0.003	0.127^*^	−0.048	−0.262^**^	-			
21. Photo quality lead entrepreneur	4.386	0.516	0.150^**^	0.055	0.046	−0.037	−0.009	0.102^*^	0.018	0.004	0.007	−0.056	0.000	0.024	0.040	−0.041	−0.013	0.044	0.001	0.056	−0.078	0.013	-		
22. Photo quality venture team	4.287	0.594	0.085	0.032	0.168^**^	−0.027	−0.027	−0.022	−0.017	0.059	0.035	−0.062	0.022	0.136^**^	−0.026	−0.038	−0.003	0.052	−0.036	0.081	−0.030	−0.047	0.572^**^	-	
23. Photo quality investor	4.433	0.512	0.029	−0.129	−0.044	0.045	−0.016	0.003	0.029	−0.034	−0.030	−0.008	0.003	−0.056	−0.044	−0.016	0.015	0.064	0.045	−0.027	−0.079	0.012	0.119^*^	0.091	-
24. Country GDP dummy	0.59	0.728	0.081	0.012	−0.067	0.001	−0.045	0.015	−0.054	−0.066	−0.044	−0.003	−0.029	−0.085	0.118^*^	0.003	0.020	−0.059	−0.016	−0.053	−0.031	0.142^**^	0.048	−0.144^**^	0.134^**^

**p < 0.01 level (2-tailed). *p < 0.05 level (2-tailed).

Table 2 exhibits the OLS regression results for first-round risk-taking. Model 1 contains the control variables. In Model 2, Lead entrepreneurs’ and VC investors’ physical attractiveness were added. In Model 3, the interaction term Lead entrepreneur PA * Lead VC investor PA was added. The results of Model 3 show significant direct and positive effects of Lead entrepreneur attractiveness [unstandardized beta (B) = 0.232, *p* = 0.001] and VC investor attractiveness (*B* = 0.138, *p* = 0.038), while the interaction term is negative and approached significance (*B* = −0.113, *p* = 0.080). Probing this interaction effect (*cf.* Hayes and Matthes, 2009) reveals that only among VCs who are below-average or average in physical attractiveness, is there a statistically significant positive relationship between lead entrepreneur PA and first round risk-taking (specifically, [*t*(309) = 3.6402, *p* = 0.0003] for VCs of below-average PA, with a 95% confidence interval (CI) from 0.1407 to 0.4750; and [*t*(309) = 2.7940, *p* = 0.0055] for VCs of average PA, with a 95% CI from 0.0585 to 0.3374). The conditional effect for VCs of above-average attractiveness is insignificant ([*t*(309) = 0.8700, *p* = 0.3850], with a CI from −0.1130 to 0.2921) (Table 3A).

**Table 2:** OLS results for first-round risk-taking.

Variables	Model 1			Model 2			Model 3		
	Beta	S.E.	Sign.	Beta	S.E.	Sign.	Beta	S.E.	Sign.
Constant	8.884	0.885	<0.001	7.460	0.936	<0.001	7.972	0.988	<0.001
Controls									
Venture age	0.402	0.059	<0.001	0.408	0.057	<0.001	0.405	0.057	<0.001
Employee growth	0.011	0.003	<0.001	0.010	0.003	<0.001	0.010	0.003	<0.001
B2B-dummy	−0.120	0.148	0.419	−0.081	0.145	0.576	−0.102	0.144	0.481
Venture team size	0.336	0.182	0.066	0.321	0.178	0.072	0.383	0.174	0.029
Venture team educational div. (level)	−0.015	0.172	0.928	−0.017	0.168	0.919	−0.014	0.167	0.932
Venture team educational div.(type)	−0.240	0.155	0.123	−0.143	0.154	0.351	−0.140	0.153	0.360
Entrepreneurial experience	0.000	0.000	0.330	0.000	0.000	0.378	0.000	0.000	0.336
Venture team physical attractiveness	−0.047	0.044	0.285	−0.046	0.043	0.280	−0.056	0.042	0.185
VC Investor team size	0.014	0.016	0.368	0.016	0.015	0.285	0.014	0.015	0.349
VC Investor team educ. Div.(level)	−0.040	0.195	0.838	−0.031	0.190	0.872	−0.018	0.190	0.923
VC Investor team educ. Div. (type)	0.119	0.195	0.541	0.025	0.192	0.896	0.026	0.191	0.892
Investor VC experience	0.013	0.019	0.487	0.015	0.019	0.437	0.016	0.018	0.391
Number of offices	0.057	0.069	0.408	0.096	0.068	0.162	0.114	0.068	0.092
Educational similarity	0.046	0.142	0.749	−0.123	0.146	0.402	−0.166	0.145	0.252
Co-ethnicity	−0.063	0.134	0.636	−0.099	0.132	0.453	−0.103	0.131	0.431
Geographic distance (log)	0.006	0.021	0.768	0.016	0.020	0.433	0.018	0.020	0.371
Photo quality lead entrepreneur	0.232	0.147	0.117	0.271	0.144	0.061	0.306	0.143	0.034
Photo quality venture team	0.164	0.130	0.207	0.124	0.128	0.333	0.109	0.126	0.376
Photo quality VC investor	−0.042	0.123	0.732	−0.042	0.120	0.729	−0.039	0.118	0.740
Country GDP dummy	0.150	0.085	0.080	0.164	0.083	0.050	0.173	0.083	0.037
Year dummies	Yes	Yes	Yes	Yes	Yes	Yes	Yes	Yes	Yes
Sub-industry dummies	Yes	Yes	Yes	Yes	Yes	Yes	Yes	Yes	Yes
Independent variables									
Lead entrepreneur Physical attractiveness				0.238	0.070	<0.001	0.232	0.070	0.001
Lead VC investor PA				0.141	0.066	0.034	0.138	0.066	0.038
Lead entrepreneur PA * Lead investor PA							−0.113	0.065	0.080
*R*	0.539			0.573			0.587		
*R* ^2^	0.291			0.328			0.344		
*F*	4.523		<0.001	4.992		<0.001	5.157		<0.001
*N*	341			341			341		

Two-tailed tests. Unstandardized beta-coefficients are reported.

**Table 3:** Results of probing analysis interaction effects.

(A) Conditional effects for focal predictor at values of the moderator variable for first-round risk-taking						
VC PA	Beta	S.E.	*t*	Sign.	LLCI	ULCI
−0.9575	0.3063	0.0842	3.6402	0.0003	0.1407	0.4720
0.0000	0.1979	0.0708	2.7939677	0.0055	0.0585	0.3374
0.9575	0.0895	0.1023	0.8700	0.3850	−0.1130	0.2921
(B) Conditional effects for focal predictor at values of the moderator variable for second-round risk-taking						
VC PA	Beta	S.E.	*t*	Sign.	LLCI	ULCI
−0.9863	−0.0239	0.0110	−2.1622	0.0320	−0.0457	−0.0021
0.0000	−0.0101	0.0089	−1.1283	0.2611	−0.0278	0.0076
0.9863	−0.0037	0.0125	0.2957	0.7679	−0.0210	0.0284

**Figure 1:**
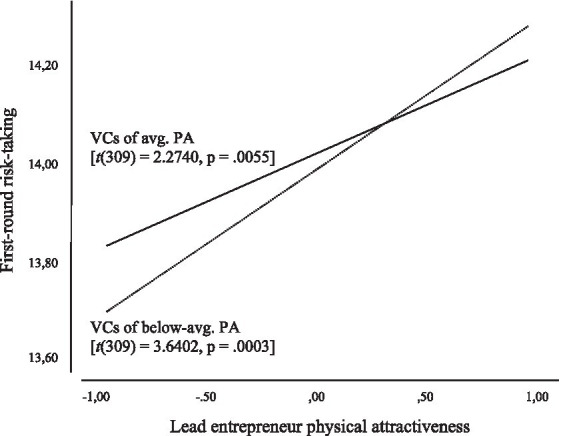
Probing results for moderating effect of VC physical attractiveness on first-round risk-taking.

These results indicate that the effect of lead entrepreneur attractiveness on the level of first-round risk-taking could be stronger for VCs of below-average attractiveness when compared to VCs of average attractiveness. The effects are plotted in Figure 1 to facilitate interpretation of results. As the figure shows, VCs of above-average attractiveness generally tend to incur more financial risks, but appear less sensitive to the attractiveness of the lead entrepreneur when compared to VCs of average attractiveness, given the steeper slope for this latter group.

**Figure 2:**
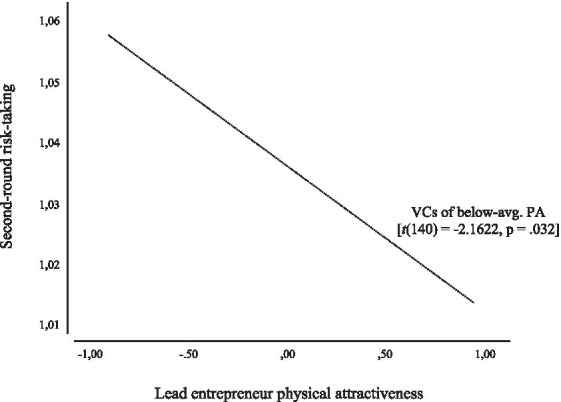
Probing results for moderating effect of VC physical attractiveness on second-round risk-taking.

Table 4 shows the regression results for second-round risk-taking. Looking at Model 3, the direct effect of Lead entrepreneur attractiveness is negative yet insignificant (*B* = −0.011, *p* = 0.199), while the direct effect for VC attractiveness is positive and insignificant (*B* = 0.007, *p* = 0.348). The interaction term is, however, positive and approached significance (*B* = 0.014, *p* = 0.073). Probing this effect reveals that only for VCs of below-average attractiveness, is there a statistically significant negative relationship between lead entrepreneur PA and second-round risk-taking ([*t*(140) = −2.1622, *p* = 0.032], with a 95% CI from −0.0457 to −0.0021) (see Table 3B). This interaction effect is plotted in Figure 2.

**Table 4:** OLS results for second-round risk-taking.

Variables	Model 1			Model 2			Model 3		
	*Beta*	*S.E.*	*Sign.*	*Beta*	*S.E.*	*Sign.*	*Beta*	*S.E.*	*Sign.*
Constant	1.108	0.129	<0.001	1.154	0.144	<0.001	1.154	0.143	<0.001
Controls									
Venture age	−0.009	0.007	0.195	−0.011	0.007	0.130	−0.010	0.007	0.143
Employee growth	0.000	0.000	0.606	0.000	0.000	0.728	0.000	0.000	0.683
B2B-dummy	−0.008	0.017	0.666	−0.007	0.017	0.702	−0.009	0.017	0.616
Venture team size	−0.004	0.021	0.862	−0.008	0.021	0.698	−0.015	0.021	0.494
Venture team educational div. (level)	−0.010	0.022	0.643	−0.014	0.022	0.525	−0.013	0.022	0.551
Venture team educational div.(type)	0.010	0.019	0.608	0.012	0.019	0.533	0.010	0.019	0.582
Entrepreneurial experience	0.000	0.000	0.150	0.000	0.000	0.157	0.000	0.000	0.145
Venture team physical attractiveness	0.004	0.005	0.415	0.005	0.005	0.292	0.007	0.005	0.177
VC Investor team size	0.001	0.002	0.473	0.001	0.002	0.613	0.001	0.002	0.494
VC Investor team educ. Div.(level)	0.028	0.025	0.266	0.032	0.025	0.212	0.025	0.025	0.327
VC Investor team educ. Div. (type)	0.012	0.026	0.653	0.018	0.026	0.499	0.024	0.026	0.351
Investor VC experience	−0.008	0.005	0.129	−0.008	0.005	0.148	−0.008	0.005	0.131
Number of offices	0.009	0.007	0.177	0.008	0.007	0.226	0.008	0.007	0.215
Educational similarity	0.019	0.018	0.288	0.021	0.018	0.232	0.027	0.018	0.134
Co-ethnicity	−0.008	0.016	0.627	−0.007	0.016	0.671	−0.010	0.016	0.534
Geographic distance (log)	0.003	0.003	0.304	0.003	0.003	0.357	0.002	0.003	0.434
Photo quality lead entrepreneur	0.012	0.020	0.530	0.010	0.020	0.622	0.010	0.019	0.613
Photo quality venture team	−0.005	0.014	0.718	−0.002	0.015	0.876	−0.005	0.015	0.739
Photo quality VC investor	−0.035	0.017	0.036	−0.038	0.017	0.025	−0.036	0.017	0.031
Country GDP dummy	−0.002	0.008	0.804	−0.001	0.008	0.874	−0.002	0.008	0.801
Year dummies	Yes	Yes	Yes	Yes	Yes	Yes	Yes	Yes	Yes
Sub-industry dummies	Yes	Yes	Yes	Yes	Yes	Yes	Yes	Yes	Yes
Independent variables									
Lead entrepreneur Physical attractiveness				−0.013	0.009	0.133	−0.011	0.009	0.199
Lead investor physical attractiveness				0.008	0.008	0.314	0.007	0.008	0.348
Lead entrepreneur PA * Lead investor PA							0.014	0.008	0.073
*R*	0.518			0.533			0.548		
*R* ^2^	0.268			0.284			0.300		
*F*	1.956		0.006	1.942		0.006	2.013		0.003
*N*	172			172			172		

Two-tailed tests. Unstandardized beta-coefficients are reported.

The regression results resonate with the two ways through which the effect of VC physical attractiveness on financial risk-taking was anticipated to manifest. With regard to the first way, VCs of above-average physical attractiveness seem to be more tolerant of risks as indicated by the larger first-round investment amounts they are comfortable to invest. This finding would be consistent with previous evidence suggesting that attractive people have more positive risk attitudes (Refaie and Mishra, 2020). With regard to the second way, the findings show that VCs of below-average attractiveness respond more strongly to the physical attractiveness of entrepreneurs. This seems in line with the notion that people of below-average attractiveness are more inclined to demonstrate compensation behavior (Chan, 2015) and as a result may be more sensitive to the attractiveness-halo (Westfall et al., 2020). We must bear in mind, however, that the weak associations for the interaction terms warrant caution when interpreting their meaning. The implications of this are further elaborated on in the discussion section.

### 4.2 Two-stage least squares analysis

In this study, an OLS regression analysis was performed in spite of the fact that the orthogonality assumption was not satisfied. As the regressor term *x* may correlate with error term *e*, the analysis could suffer from endogeneity. Therefore, an additional two-stage least squares analysis (2SLS) on the main effect for first-round risk-taking was performed to assess endogeneity. 2SLS was performed by making use of an instrument variable.

Entrepreneur’s pattern hair loss (PHL) was selected as endogenous source of variance and, thus, represents the instrument variable. PHL is the most common type of hair loss among men and strongly correlates with attractiveness (Van der Donk et al., 1994; Muscarella and Cunningham, 1996; Williamson et al., 2001). Hair loss has psychosocial effects by making people more self-conscious and dissatisfied with their appearance (Girman et al., 1998; Budd et al., 2000; Alfonso et al., 2005; Cash, 2009), potentially causing negative self-image and lower self-esteem. People with high self-esteem and self-confidence are typically viewed as more attractive (Langlois et al., 2000). Taking these psychosocial consequences of PHL into account, pattern hair loss may indirectly influence VC decision-making through attractiveness. As such, the selected instrument satisfies the relevance requirement that there should be a theoretical association between the instrument and the independent variable. It is also reasonable to assume that an entrepreneur’s PHL does not directly impact VC decision-making, but rather operates through other factors, such as attractiveness. This implies that PHL meets the exclusion restriction criterion^2^ as well. Moreover, genetically determined qualities (such as testosterone level, which is strongly associated with PHL) tend to be suitable instruments (Antonakis et al., 2014).

Entrepreneurs’ PHL was estimated by means of their LinkedIn profile photos using the universal BASP classification system developed by Lee et al. (2007). The BASP classification system specifies gradually developing patterns of hair loss. As entrepreneurs’ LinkedIn profile pictures involved frontal portraits, the L-type, M-types, C-types, U-types, and F-types PHL could be used to determine anterior hairline shapes and hair density [see Lee et al. (2007) for visualizations of PHL-types]. V-types PHL could not be identified since these involve hair loss on the back of someone’s head. Subsequently, a 6-point scale was developed to categorize sampled lead-entrepreneurs’ degree of hair loss: 1 = L; 2 = M0, C0, F1; 3 = M1, C1, F2; 4 = M2, C2, F3; 5 = M3, C3; 6 = U1, U2, U3.

Table 5A contains the first-stage regression results for lead entrepreneur physical attractiveness. The instrument (PHL) is the main independent variable and all original control variables were included. Table 5A shows that, as expected, the coefficient estimate for PHL is negatively associated with physical attractiveness (ß = −0.109, *p* = 0.004). Table 5B contains the second-stage regression results, with *first-round risk-taking* as the dependent variable. The coefficients for the 2SLS regression (Table 5B) are generally consistent with the original estimates (Table 2, Model 2). The magnitudes of the 2SLS coefficients for the independent variables suggest that the original OLS regression estimates for physical attractiveness and co-ethnicity were slightly underestimated. Nonetheless, the general interpretation of the results remains unaffected.^3^

**Table 5:** Results of 2SLS-analysis.

(A) First-stage regression for lead entrepreneur physical attractiveness (*N* = 341)			(B) Second-stage regression for first-round risk-taking (*N* = 341)		
Variable	Coeff.	Sign.	Variable	Coeff.	Sign.
Constant	3.694	<0.001	Constant	12.242	<0.001
Controls			Controls		
Venture age	−0.027	0.537	Venture age	0.328	<0.001
Employee growth	0.002	0.457	Employee growth	0.012	0.001
B2B-dummy	0.021	0.851	B2B-dummy	−0.054	0.796
Venture team size	0.105	0.453	Venture team size	0.454	0.076
Venture team educational diversity (level)	0.035	0.790	Venture team educational diversity (level)	0.007	0.975
Venture team educational diversity (type)	−0.290	0.015	Venture team educational diversity (type)	−0.512	0.064
Entrepreneurial experience	0.000	0.945	Entrepreneurial experience	0.000	0.499
Venture team PA	−0.015	0.655	Venture team PA	−0.057	0.353
VC Investor team size	−0.013	0.279	VC Investor team size	−0.005	0.825
VC Investor team educ. Div. (level)	0.208	0.158	VC Investor team educ. Div. (level)	0.170	0.565
VC Investor team educ. Div. (type)	0.286	0.052	VC Investor team educ. Div. (type)	0.474	0.158
Investor VC experience	0.004	0.789	Investor VC experience	0.023	0.399
Number of offices	−0.142	0.008	Number of offices	−0.081	0.532
Educational similarity	0.652	<0.001	Educational similarity	0.751	0.092
Co-ethnicity	−0.053	0.605	Co-ethnicity	−0.143	0.451
Geographic distance (log)	−0.031	0.045	Geographic distance (log)	−0.024	0.491
Photo quality lead entrepreneur	−0.136	0.235	Photo quality lead entrepreneur	0.109	0.623
Photo quality venture team	0.259	0.010	Photo quality venture team	0.452	0.061
Photo quality investor	−0.073	0.441	Photo quality investor	−0.121	0.495
Country GDP dummy	−0.025	0.699	Country GDP dummy	0.139	0.247
Year dummies	Yes	Yes	Year dummies	Yes	Yes
Sub-industry dummies	Yes	Yes	Sub-industry dummies	Yes	Yes
Lead investor physical attractiveness	0.054	0.295	Lead investor physical attractiveness	0.202	0.042
Instrument			Independent variable		
Pattern hair loss	−0.109	0.004	Lead entrepreneur Physical attractiveness	0.301	0.021

Two-tailed tests. Unstandardized coefficients are reported.

## 5 Discussion and conclusion

The current study explored the role of VCs’ physical attractiveness and their early-stage funding of new, high-technology ventures. In so doing, this study departed from Chan’s, 2015 experimental investigations of same-sex stimuli and financial risk-taking as well as Franke et al.’s (2006) suggestion to explore biases and heuristics in the context of stage financing. The findings suggest that the halo-effect that has been found to influence VCs’ decision-making (e.g., Baron et al., 2006; Brooks et al., 2014) could be conditional on VCs’ own physical attractiveness. These findings both corroborate and extend Chan’s (2015) seminal experiments on same-sex stimuli and financial risk-taking. The current study provides initial corroborating evidence by showing the presence of upward social comparison effects of male VCs to attractive male entrepreneurs, thereby demonstrating the relevance of same-sex stimuli on financial risk-taking in a naturalistic setting. However, this effect was only established for VCs of average and below-average attractiveness, and not for VCs of above-average attractiveness.

By addressing the role of same-sex stimuli in the context of staging, this study also extends current theory as it considers upward social comparison effects beyond initial encounters between individuals. In so doing, the findings suggest that same-sex stimuli effects could linger on beyond initial interpersonal meetings, and may change during later stages of the (investment) relationship. Specifically, the effect of entrepreneurs’ physical attractiveness on VCs’ risk-taking changed from positive to negative for VCs of below-average attractiveness as the funding process progressed. One explanation could be the occurrence of a delayed beauty penalty effect (Li and Zhou, 2014). It is quite imaginable that highly attractive entrepreneurs who do not deliver on expectations, receive a beauty penalty when a VC’s initial expectations were affected by the entrepreneur’s attractiveness. The beauty penalty effect has not, however, been shown before in a context of same-sex stimuli and financial risk-taking. Other mechanisms could be at play as the nature of the risk-taking could change with every new funding round (Tian, 2011). Nonetheless, the results suggest a complex and intriguing interplay between VCs own physical attractiveness and the attractiveness of the entrepreneurs they have chosen to invest in.

While this study’s findings are informative, several unanswered questions remain. First, as this study relied on a combination of archival and observational data, no data were available on VCs’ individual psychological dispositions or characteristics. Dispositions such as loss aversion, risk tolerance, and overconfidence (Hambrick and Mason, 1984; Kahneman, 2011) and characteristics such as self-esteem could play a role in VCs’ financial risk-taking, and therefore should be considered in future research. Second, the limited number of women in the initial sample of ventures prohibited a statistical analysis of the role of same-sex stimuli and financial risk-taking among females. Future research may address this question in the context of staging as well. The share of women among both IT startups and VC investors is increasing (Atomico, 2020), which could make the investigation of same-sex stimuli among female entrepreneurs and VCs more pertinent (Brooks et al., 2014; Balachandra et al., 2017). The study of same-sex stimuli in the context of female entrepreneurs and female VCs could be worthwhile, because women have faced less intrasexual competition than men. As a result, different effects on female VC risk-taking and associated staging considerations can be expected (Chan, 2015; Friedl et al., 2020). Third, while the VC setting is a highly suitable context to study financial risk-taking behaviors, we must bear in mind that the type of financial risk-taking in the VC setting differs somewhat from the type of financial risks discussed in Chan (2015). Specifically, VCs may incur substantial losses, while in Chan’s experiments the risk revolved around certain (yet incremental) versus uncertain (yet substantial) gains. Another notable difference is that each subsequent round of investment essentially increases the total financial risk for the VC. This could imply that additional theoretical frameworks can be utilized to further improve our understanding of financial risk-taking in same-sex constellations. In addition, other research settings may be considered to further corroborate and extend our understanding of the role of same-sex stimuli. To start with, as this study focused on staging, other studies may consider the role of same-sex stimuli effects during the preceding selection and screening phases. To date, studies of VC selection decisions have centered their attention on the physical attractiveness of the entrepreneur, but neglected the attractiveness of the VC investor him- or herself. Future studies may also consider other settings that are characterized by substantial financial risk-taking and that involve two parties that need to develop a trusting relationship. For instance, CEOs and TMT-members are confronted with substantial risks when considering an alliance with another company, and work intensively with representatives from that other company when negotiating the alliance (Lau and Murnighan, 1998). Fourth, the attractiveness ratings were based on unstandardized profile photos of entrepreneurs and VCs, and it wasn’t possible to determine how recent these photos were. While no significant correlation was detected between photo quality and attractiveness of the lead entrepreneurs and the VCs (Table 1), the use of standardized profile photos is preferred. Moreover, even though the use of still photos have been found suitable to estimate a target’s physical attractiveness (Rhodes et al., 2011), they cannot be used to estimate other relevant attributes that could interact with attractiveness (e.g., tone of voice, height, body movement). This would require the use of video captures or real-life observations. Fifth and final, this study demonstrated that same-sex stimuli affect financial risk-taking beyond the initial encounter. Yet, more research is needed to determine how robust these findings are, how long such effects linger on, and what shape they take over time. Particularly, the encountered conditional effects for VCs of average and below-average attractiveness may be considered as a first indication of the relevance of same-sex effects on male financial risk-taking in the VC financing context, but also should be interpreted with caution. Future research is needed to assess the robustness of these interaction effects, preferably with larger samples, in other contexts, and using alternative methods.

To conclude, this study took Chan’s experimental study of same-sex stimuli and financial risk-taking as point of departure, and sought to corroborate its findings in a naturalistic setting while generating novelty by considering later-stage relationships. Its findings give ample reason to further investigate the phenomenon, especially since same-sex encounters are abundant across both personal and professional settings.

## Data availability statement

The raw data supporting the conclusions of this article will be made available by the authors, without undue reservation.

## Author contributions

MB: Writing – original draft.

## Footnotes

^1^Both types of educational diversity were calculated using Blau’s index of variability (Blau, 1977). For diversity in educational level, the following categories were used: PhD; Master’s; Bachelor’s; High-school. For diversity in educational background, entrepreneurs’ study backgrounds were categorized as follows: Business & Economics; Engineering; Humanities & Arts; Law & Political Science; Science.

^2^In an additional analysis, the instrument variable was regressed against the dependent variable for first-round risk-taking. To this end, it replaced the variable for entrepreneurs’ physical attractiveness while keeping all of the original controls. The instrument variable did not directly affect the dependent variable (*ß* = 0.070, *p* = 0.141) Results are available upon request.

^3^In addition to the 2SLS-analysis, a Coarsened Exact Matching (CEM) procedure was carried out to further substantiate the inferential claims of this study. Particularly, “statistical twin groups” were created based on VC physical attractiveness, VC experience, and VC educational similarity. The CEM findings suggested that, *ceteris paribus*, VCs of above-average attractiveness took more first-round and second round risks compared to VCs of below-average attractiveness.
